# Supplementary material for: Multiple sources of slow activity fluctuations in a bacterial chemosensory network
Source: eLife. 2017 Dec 12;6:e26796. doi: 10.7554/eLife.26796 (PMC5809148; doi:10.7554/eLife.26796)
Supplement: Supplementary file 1 [file elife-26796-supp1.docx]

**Supplementary File S1. List of strains and plasmids used in the study**

Table S1. Strain list

| Strain | Genotype | Background | Ref. |
| --- | --- | --- | --- |
| VS181 | Δ(tar,tsr,trg,tap,aer) Δ(cheY,cheZ) | RP437 | [1] |
| VH1 | Δ(tar,tsr,trg,tap,aer) Δ(cheY,cheZ) Δ(cheR,cheB) | RP437 | [2] |
| VF7 | Δ(tar,tsr,trg,tap,aer) Δ(cheY,cheZ) Δ(cheR,cheB) cheW(R117D,F122S) | RP437 | [3] |
| VF8 | Δ(tar,tsr,trg,tap,aer) Δ(cheY,cheZ) cheW(R117D,F122S) | RP437 | [3] |

Table S2. Plasmid list

| Plasmid | Genotype | Antibiotic | Induction | Ref. |
| --- | --- | --- | --- | --- |
| pVS88 | cheY-YFP/cheZ-CFP,  pTRC99a derivative | Ampicillin 100µg/ml | IPTG 200 µM, | [1] |
| pVS1092 | Tar^QEQE^,  pKG110 derivative | Chloramphenicol  17 µg/ml | Salicylate 2 µM,  If not otherwise stated | [1] |
| pVS1087 | Tar^QEEE^,  pKG110 derivative | Chloramphenicol  17 µg/ml | Salicylate 2 µM,  If not otherwise stated | [4] |
| pVS1086 | Tar^EEEE^,  pKG110 derivative | Chloramphenicol  17 µg/ml | Salicylate 2 µM,  If not otherwise stated | [5] |

[1] Sourjik V. and Berg H. C. *Functional interactions between receptors in bacterial chemotaxis.*  Nature (2004) **428**, 437, doi:10.1038/nature02406

[2] Endres R. G., Oleksiuk O., Hansen C. H., Meir Y., Sourjik V. and Wingreen N. S. *Variable sizes of Escherichia coli chemoreceptor signaling teams.*  Molecular Systems Biology (2008) **4**, 211, doi:10.1038/Msb.2008.49

[3] Frank V., Pinas G. E., Cohen H., Parkinson J. S. and Vaknin A. *Networked Chemoreceptors Benefit Bacterial Chemotaxis Performance.*  MBio (2016) **7**, e01824, doi:10.1128/mBio.01824-16

[4] Neumann S., Grosse K. and Sourjik V. *Chemotactic signaling via carbohydrate phosphotransferase systems in Escherichia coli.*  Proceedings of the National Academy of Sciences of the United States of America (2012) **109**, 12159, doi:10.1073/pnas.1205307109

[5] Krembel A., Colin R. and Sourjik V. *Importance of Multiple Methylation Sites in Escherichia coli Chemotaxis.*  PLoS One (2015) **10**, e0145582, doi:10.1371/journal.pone.0145582
